# Electrochemical biosensing of alpha-fetoprotein based on carboxylated multi-walled nanotube-polyAzur A (in DES)-gold nanoparticles

**DOI:** 10.55730/1300-0527.3797

**Published:** 2026-04-10

**Authors:** Merve YILMAZ ÇILÇAR, Melike BİLGİ KAMAÇ

**Affiliations:** Department of Chemistry, Faculty of Science, Çankırı Karatekin University, Çankırı, Turkiye

**Keywords:** Alpha-fetoprotein biosensor, nanostructures, deep-eutectic solvent, polyAzur A, cancer biomarker

## Abstract

AFP, a cancer biomarker, is monitored during fetal development and in the serum of pregnant women. Sensitive and rapid monitoring of AFP is crucial for effective management. In this study, screen-printed carbon electrodes (SPCEs) modified with multi-walled carbon nanotubes (MWCNT-COOH), polyAzur A (PAA), which was prepared in a deep eutectic solution (DES), and gold nanoparticles (AuNP) were fabricated. AFP biosensors were developed for practical, sensitive, low-cost, and rapid detection of AFP. Optimizations of nanostructures (MWCNT-COOH, AuNP) and polymers (PAA_DES_) were performed. The MWCNT-COOH, PAA_DES,_ and AuNP-modified SPCEs were characterized by FE-SEM-EDX, FTIR, and XRD techniques. The amounts of 6-MOH, APTES, GA, and anti-AFP used to prepare AFP biosensors and the times for anti-AFP and AFP were also optimized. The linear range and detection limit of the AFP biosensors were established as 0.5–250 ng/mL and 0.15 ng/mL, respectively. Stability and shelf life were monitored for 60 days and 24 weeks, respectively. The AFP biosensors exhibited high selectivity (97.5%) for AFP in the presence of other cancer biomarkers, including cancer antigen 125 and human epididymis protein 4. Reproducibility tests of the AFP biosensors demonstrated responsiveness across five replicates (RSD: 4.80%, n = 5). Although designed to be disposable, the developed AFP biosensors were reusable up to two times. AFP levels in commercially obtained blood serum were measured by DPV for 2 minutes using AFP biosensors. The developed AFP biosensors are potential candidates for use in point-of-care testing.

## 1. Introduction

Alpha-fetoprotein (AFP) is a protein of approximately 70 kilodaltons that belongs to the albuminoid gene family; the AFP gene is located on the q arm of chromosome 4 [1]. AFP is a Food and Drug Administration (FDA)-approved biomarker for the diagnosis and monitoring of germ cell tumors [2]. AFP levels in the serum of newborn infants are approximately 17–44 ng/mL and decrease to 10–15 ng/mL with age [3,4]; they increase in cases of immunodeficiency [5]. AFP is widely used as a biomarker for the diagnosis of various cancers (hepatocellular carcinoma, endodermal tumors, ovarian and gastric cancers) [6–7]. Therefore, accurate and sensitive determination of AFP is crucial. Conventional methods for AFP determination, including radioimmunoassays [8–9], enzyme-linked immunosorbent assays [10–11], fluorescence immunoassays [12–13], and electrochemiluminescent immunoassays [14–15], are highly sensitive but require specialized equipment, lengthy experimental procedures, and entail high costs. The disadvantages of these traditional approaches to early diagnosis include their time-consuming nature, which hinders rapid diagnosis in clinical settings [16]. Rapid, affordable, and simple methods with high sensitivity for detecting AFP are crucial for cancer research [17]. Electrochemical biosensors offer all these features [18–21].

Most biosensors designed for biomarker detection employ a sandwich format, which requires two different antibodies to bind specifically to the antigen [22]. These biosensors use magnetic micro- and nanoparticles, nanomaterials, and conductive polymers to enhance signals or enrich biomolecules [23–24]. Despite their high accuracy and selectivity, the preparation of labeled AFP biosensors reported in the literature is costly and time-consuming [25]. Label-free electrochemical biosensors are more practical and less costly than labeled biosensors. In the preparation of label-free AFP immunosensors, materials such as MnO_2_-functionalized mesoporous carbon hollow sphere composite [26], reduced graphene oxide (RGO), polyneutral red, AuNPs [18], hybrid nanocomposite of carbon black and palladium nanoparticles [27], crown-like structure forming potassium hexacyanoferrate, copper nanoparticles, and carboxyl-functionalized reduced graphene oxide [28] were used. The use of nanomaterials and polymers has improved the parameters of biosensors, including stability (particularly storage stability) and reproducibility [18]. In recent years, AFP biosensors prepared by incubating GCEs modified with the RGO@platinum nanoparticles/molybdenum disulfide composite overnight, with anti-AFP, have the disadvantage of requiring time-consuming antibody immobilization [29]. AFP biosensors were developed using indium tin oxide (ITO) treated with hydroxyl groups and silane reagent 3-(trimethoxysilyl)propyl methacrylate. However, the triple electrode system used is not suitable for point-of-care testing (POCT) [30]. AFP biosensors were prepared using GCEs modified with nitrogen-sulfur-iron (III) oxide carbon nanodots (Fe_2_O_3_@NS-CDs). It has been reported that Fe_2_O_3_@NS-CDs exhibit electrochemical conductivity, increasing the surface area of the electrode and the anti-AFP capture capacity of the surface groups [31]. The materials used to build the aforementioned electrochemical biosensors enhanced their electrical conductivity by increasing the surface area of the electrodes and facilitating electron transfer.

Carbon-based nanomaterials such as RGO [32–33], MWCNT-COOH [34], fullerene (C60) [35], graphene oxide (GO) [36], RGO [18,37–38], and MXene [39–40] are frequently used to enhance the surface area of electrochemical biosensors. Metal nanomaterials, such as gold, silver, copper, and platinum, are frequently preferred in electrochemical biosensors due to their high electrical conductivity and biocompatibility [17]. Among these nanometals, AuNPs exhibit synergistic effects when combined with conductive polymers and possess excellent electrical conductivity [41]. In addition to nanomaterials, conductive and redox polymers are frequently used in the design of electrochemical biosensors due to their high conductivity [42–44]. Recently, deep eutectic solvents (DES), employed in green methodologies, have attracted interest for the preparation of these polymers. DESs, prepared rapidly using hydrogen bond acceptors and hydrogen bond donors, exhibit low toxicity and high mechanical strength. DESs are gradually replacing other organic solvents [21,45]. Conductive polymers prepared in DES are used in the production of sensors [46–49] and biosensors [21].

In recent years, azine dyes prepared in DES have attracted attention. For example, in a study conducted by our team, TB, an azine monomer prepared in DES, was electropolymerized onto SPCE, modified with AuNPs, and used in biosensors for the analysis of cancer antigen 125 (CA125). PTB prepared in DES produced a synergistic effect with AuNPs, thereby increasing the analytical performance of the biosensor by expanding the electrode surface area [25]. In another study by our team, SPCEs were modified with zinc oxide nanorods (ZnONR), polymethylene blue (PMB), and AuNPs, prepared in DES, and were used for the electrochemical determination of serotonin. ZnONR, PMB, and AuNP exhibited a synergistic effect, amplifying the electrochemical signal and enhancing the sensor’s performance [46]. Also, in another study by our team, SPCEs were modified with titanium oxide nanoparticles (TiO_2_NP), AuNP, and polynile blue (PNB) prepared in DES and utilized for the simultaneous determination of dopamine and serotonin. TiO_2_NP, AuNP, and PNB_DES_ have been reported to show high performance in electrochemical analysis [47]. Azur A (AA), an azine dye, is also electropolymerized, as is TB [50]. and used as a conductive polymer in electrochemical sensors and biosensors [51]. PolyAzure A (PAA) is used to fabricate sensors for glucose [52], hemoglobin [53], DNA [54], nicotinamide adenine dinucleotide [55], and hydrogen peroxide [56–57]. These studies emphasized that polymer conductivity increases the electroactive surface area of the electrode, thereby enhancing the sensor signal. The use of PAA in the production of sensors and biosensors is believed to enhance detection performance. In this study, we electrochemically polymerized AA in DES on SPCE for the first time and investigated its application in biosensors.

Prompt, precise identification of biomarkers, such as AFP, is essential for the early detection and treatment of diverse diseases, including cancer, amid numerous global health challenges. In this study, we created fast, easy-to-use, disposable, and low-cost AFP immunosensors by modifying SPCEs with MWCNT-COOH, PAA_DES_, and AuNP. The amount of conductive redox polymer and nanomaterials on the SPCE surface, and the operating parameters of the AFP immunosensors were optimized. The study examined MWCNT-COOH, PAA_DES_, and AuNPs, both individually and in combination, and found that when used together, they enhanced conductivity and electroactive surface area. These nanostructures and polymers demonstrated superior performance on key parameters, including reproducibility, stability, and shelf life, for AFP immunosensors. The performance of the AFP immunosensor, designed for POCT, was also evaluated in real samples. AFP levels in blood serum were measured using the DPV method within two minutes.

## 2. Experimental Section

**Figure 1:**
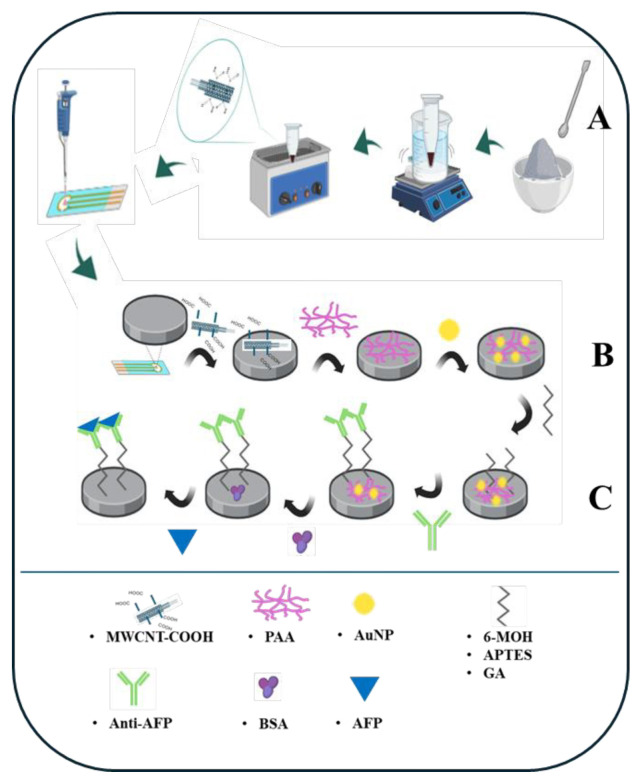
Depicts the preparation of the MWCNT-COOH (A), the construction stage of the modified electrodes (B), and the AFP immunosensor (C).

All materials, reagents, and instruments used; electrochemical measurement parameters; solution and modified-electrode preparation procedures; and AFP biosensor fabrication details are available in the Supplementary Material file. The preparation scheme for the modified electrode and the fabrication of the AFP biosensor are given in Figure 1A-C.

## 3. Result and Discussion

### 3.1. MWCNT-COOH modification on the SPCEs

Physical adsorption is a practical and straightforward method for modifying electrodes with nanoparticles (NPs) [43,47]. When there are insufficient NPs on the electrode surface, sufficient conductivity may not be achieved [43,58]. When the amount is high, it causes an elevated non-faradaic current, which directly affects the detection limit and reduces sensitivity [43,59]. For this reason, optimization of the amount of NM is essential. For the optimization of the amount of MWCNT-COOH on SPCEs, CV measurements were applied at varying scan rates (10–125 mV s^−1^) in the redox probe solution of the MWCNT-COOH-modified SPCEs prepared with varying numbers of layers (0–1-2–3-4-LBL). Voltammograms and Ipa-v^1/2^ graphs are given in Figure S1A-J. The electroactive surface areas (Aea) of the MWCNT-COOH modified SPCEs were computed (using the Randles-Sevcík equation *Ip*=2.69×10^5^ *n*^3/2^*AeaC√Dϑ*, [60]. The Aea values of the modified electrodes were calculated to be 0.1252 cm^2^ (0-LBL), 0.1342 cm^2^ (1-LBL), 0.1775 cm^2^ (2-LBL), 0.2005 cm^2^ (3-LBL), and 0.1581 cm^2^ (4-LBL). The highest Aea value was obtained with the electrode prepared with 3 LBL MWCNT-COOH. In 1-LBL and 2-LBL MWCNT-COOH-modified electrodes, sufficient electrochemical conductivity was not achieved, and electrochemical conductivity and electron transfer were insufficient compared to 3-LBL [61]. Accordingly, the Aea values for 1-LBL and 2-LBL are lower than those for 3-LBL. 4 LBL MWCNT-COOH formed a thicker layer than 3 LBL, and the Aea value decreased. This decrease is thought to result from a thick surface layer that prevents electron transfer. Based on these results, the optimal MWCNT-COOH amount was 3-LBL, since the SPCE/MWCNT-COOH (3-LBL) electrode exhibited the highest conductivity.

### 3.2. AA_DES_ electropolymerization on the SPCE/MWCNT-COOH

In this part of the study, electropolymerization of AA_DES_ on SPCE/MWCNT-COOHs was carried out. The CVs obtained during electropolymerization are included in Figure S2A. In the voltammograms, the monomer’s oxidation peak in the first cycle appears at approximately -0.262 V, and its reduction peak appears at approximately -0.332 V. In our team’s previous studies with azine polymers, oxidation peaks of the monomers were recorded at approximately -0.130 V and -0.100 V, and reduction peaks were recorded at approximately -0.120 V and -0.190 V in the first cycles of PMB_DES_ and PTB_DES_ polymers, respectively [46,47]. Porfireva et al. applied electropolymerization of AA to GCEs and observed redox peaks corresponding to the monomer (Epa: −0.170 V, Epc: −0.220 V) [54]. When the potential reaches approximately +0.9 V, cationic radicals of the monomer are constituted and react with other AA_DES_ monomers, initiating AA_DES_ polymerization [46–47,56,62–63]. In the literature, the electropolymerization of AA (in aqueous phosphate solution) was carried out on a GCE, and the cationic radical of the monomer was monitored at potentials ranging from +0.8 to +0.9 V [54–56]. In our study, the oxidation peak of PAA_DES_ is observed at approximately −0.128 V (Epa) and the reduction peak at approximately −0.246 V (Epc) starting from the 2nd cycle. Gao et al. reported increasing PAA peaks at approximately 0.1 V (Epa) and −0.07 V (Epc) in the 2nd cycle [55]. Porfireva et al reported that they observed PAA peaks at −0.13 V and +0.14 V [54]. The investigations revealed that the anodic and cathodic peak potentials increase with the number of cycles. As the number of cycles increases, Epa and Epc increase and shift toward more positive values. Şahin and Ayrancı carried out the electropolymerization of polyneutral red (PNR) on SPCE/MWCNT-COOH and reported that a pH change occurred on the electrode surface due to the production of H+ that the electropolymerization to shift to a positive potential [43]. Polymerization occurs when the monomer forms cationic radicals that attack other AA_DES_ monomers, initiating polymerization. The cationic radical is then covalently linked to another aromatic ring of AA_DES_ via the amine groups via the carbon atom at the ortho position of the monomer. Thus, the electrochemical polymerization of AA proceeds via nitrogen bridges. The polymerization mechanism of PAA_DES_ is like that of PTB_DES_ [21] and PMB_DES_ [46–47,53–54].

Optimizing the film thickness of the polymer is a crucial step, as it directly affects the properties of the biosensor, including conductivity and biomolecule binding capacity. If the polymer film is too thin, the desired functionality cannot be achieved; if it is too thick, sufficient electron transfer cannot occur [43,46–47]. The optimum number of loops must be determined to ensure maximum signal transmission. To determine the optimum number of cycles, PAA_DES_ 20, 25, and 30 cycle CV was applied on SPCE/MWCNT-COOHs, and the recorded CVs are given in Figure S2A-C. A layer was formed on the surface with each PAA_DES_ cycle, and the polymer was coated on the surface. CVs of PAA_DES_-modified electrodes (in redox probe solution) were taken at different scan rates (10–125 mV s^−1^) in other cycles. Voltammograms and Ipa-v^1/2^ plots are given in Figure S3A-F. The Aea values of the modified electrodes were determined as 0.2120 cm^2^, 0.1983 cm^2^, and 0.1922 cm^2^ for 20, 25, and 30 cycles of PAA_DES_, respectively, and the highest Aea value was obtained with 20 cycles of PAA_DES_. It is believed that the polymer film on the electrodes after 25 and 30 cycles is too thick to provide the desired electron transfer. According to these results, the optimum PAA_DES_ was determined to be 20 cycles. No PAA polymer prepared has been reported in the literature, and it was prepared for the first time in our study.

### 3.3. Morphological and chemical characterizations of modified electrodes

**Figure 2:**
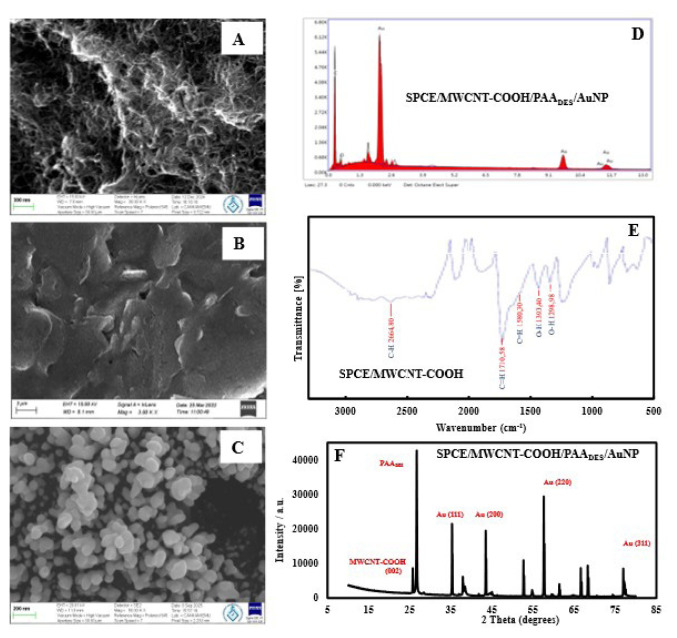
SEM images of SPCE/MWCNT-COOH (A), SPCE/MWCNT-COOH/PAA_DES_ (B), SPCE/MWCNT-COOH/PAA_DES_/AuNP (C), EDX spectra of SPCE/MWCNT-COOH/PAA_DES_/AuNP (D), FTIR spectra of SPCE/MWCNT-COOH (E), XRD spectra of SPCE/MWCNT-COOH/PAA_DES_/AuNP (F).

Morphological characterization of the modified electrodes was performed using SEM, while chemical characterization was conducted using EDX, FTIR, and XRD. SEM images of SPCE/MWCNT-COOH, SPCE/MWCNT-COOH/PAA_DES_, and SPCE/MWCNT-COOH/PAA_DES_/AuNPs are shown in Figure 2A-C, respectively. In Figure 2A, the fiber-like filamentous images were observed, providing evidence of the presence of MWCNT-COOH [64]. In Figure 2B, irregularly distributed block structures and a rougher surface overlap are observed, and these images show the polymer layers of PAA_DES_ [46–47,65]. In Figure 2C, numerous spherical nanoparticles were observed on the surface of the polymer layers [21,46–47,66]. These nanoparticles are AuNPs. In Figure 2D, the presence of the Au element is seen in the EDX spectrum of the SPCE/MWCNT-COOH/PAA_DES_/AuNP electrode. The presence of Au confirms the deposition of AuNPs on the WE [21,46–47]. According to the FTIR spectrum of MWCNT-COOH in Figure 2E, the peak at 2664.80 cm^−1^ originates from the C-H asymmetric vibration. The peaks at 1710.58 cm^−1^, 1580.30 cm^−1^, 1393.40 cm^−1^, and 1298.98 cm^−1^ are due to the vibration of the bonds in C=O and −OH in the resonance structure of the carboxyl group [67]. In the XRD spectrum of the SPCE/MWCNT-COOH/PAA_DES_/AuNP electrode in Figure 2F, a sharp diffraction peak is seen at 26.55° for PAA_DES_ [46]. Azine polymers, such as PAA, have been reported in other studies to peak at approximately 26°C [21,46,68]. A typical diffraction peak (002) at 26.45° for MWCNT-COOH [69] and four characteristic diffraction peaks (111), (200), (220), and (311) at 35.38°, 43.62°, 57.81°, and 77.24° for AuNP were observed, respectively [21,46,47].

### 3.4. Electrochemical performances of MWCNT-COOH, AA_DES,_ and AuNP modified electrodes

**Figure 3:**
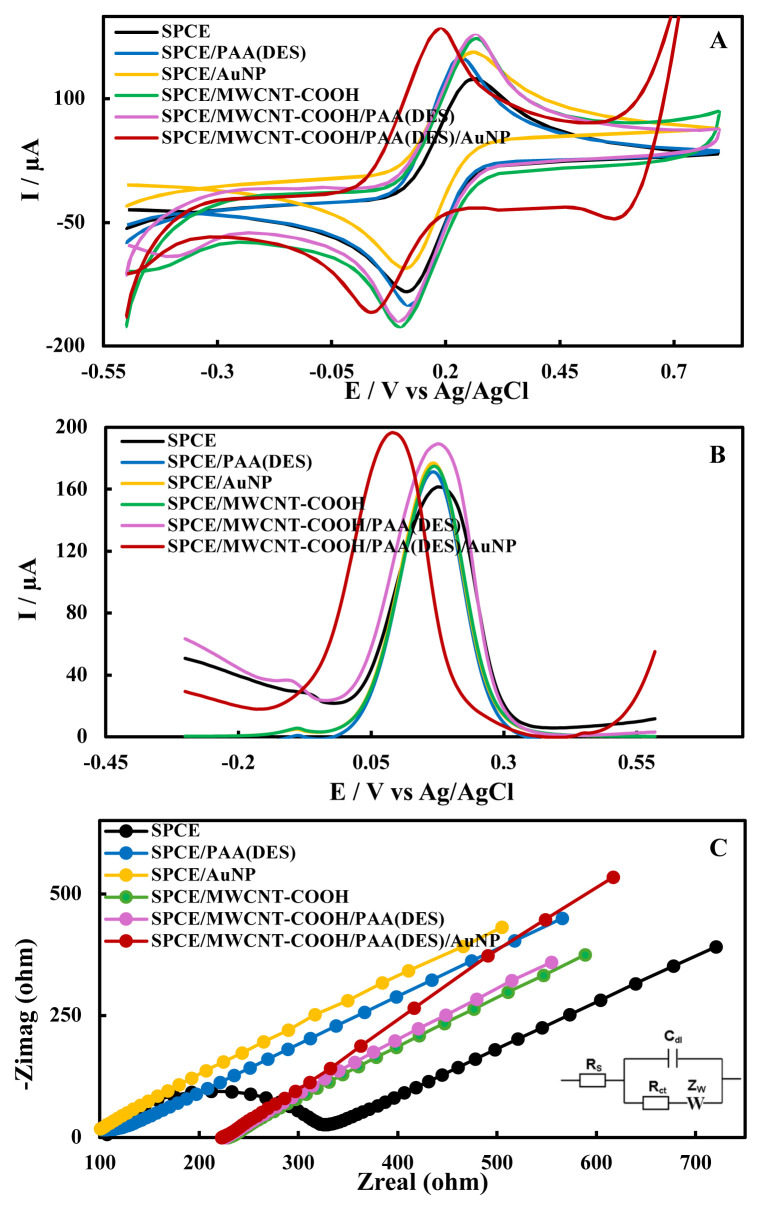
CVs (A), DPVs (B), and EISs (C) of SPCE, SPCE/PAA_DES_, SPCE/AuNP, SPCE/MWCNT-COOH, SPCE/MWCNT-COOH/PAA_DES_, SPCE/MWCNT-COOH/PAA_DES_/AuNP electrodes.

The electrochemical properties of electrodes modified with various nanoparticles and conductive polymers directly affect biosensor performance. In our study, CV, DPV, and EIS measurements were performed in the redox probe solution to investigate the electrochemical performance of MWCNT-COOH, AA_DES_, and AuNP modified SPCEs. CVs, DPVs, and EISs are given in Figure 3A-C, and Ipa_avg_ and Rct_avg_ are presented in Table S1. When examining the CV and DPV of bare SPCE, the Ipa_avg_ values are 128.62 μA and 147.89 μA. After individual PAA_DES_, AuNP, and MWCNT-COOH modifications on the SPCE, the Ipa_avg_ values obtained from CV and DPV are close to each other. These conductive materials are used in electrochemistry to enhance the Aea of the sensor and improve the biosensor’s performance. In our study, we investigated changes in the electrode’s electrochemical performance when this nanomaterial and this conductive polymer were used together. The Ipa_avg_ were further increased after PAA_DES_ (CV: 162.59 μA and DPV: 177.0 μA) and AuNP modifications (CV: 164.66 μA and DPV: 187.17 μA) on the SPCE/MWCNT-COOH electrode (CV: 155.79 μA and DPV: 172.65 μA). PAA_DES_ has electronic conductivity due to the π-conjugated structure in its aromatic ring. MWCNT-COOH possesses carboxyl functional groups and hydrophobic sidewalls comprised of sp^2^ carbons, together with a π-conjugated structure. Consequently, π-π electronic and hydrophobic interactions transpired between MWCNT-COOH and PAA_DES_. Additionally, AuNPs enhance the electrochemical conductivity by forming a bridge between MWCNT-COOH and PAA_DES_ [43,70]. Additionally, AuNPs are the nanomaterials that should be used to form regular SAMs on them. These results in CV and DPV prove that the combined use of MWCNT-COOH, PAA_DES,_ and AuNP shows a synergistic effect. SPCE/MWCNT-COOH/PAA_DES_/AuNP electrode has the best formulation. In EIS, the Rct_avg_ value decreased, and conductivity increased with PAA_DES_ and AuNP modifications (SPCE/PAA_DES_:76.70 SPCE/AuNP:61.73 ohm). The Rct value increased to 229.20 ohms with the MWCNT-COOH modification. This significant increase is due to the repulsion between the electroactive groups in MWCNT-COOH and the charged groups in the redox probe solution [71]. The modification of PAA_DES_ onto SPCE/MWCNT-COOH resulted in a decrease in the Rct_avg_ (213.90 ohm). This decrease is attributed to the interaction between the negatively charged groups in MWCNT-COOH and the redox couple in PAA_DES_, resulting in a synergistic effect that enhances electronic conductivity [43]. In the modification of SPCE/MWCNT-COOH/PAA_DES_ with AuNPs [19–20,46–47,70], the electrode’s conductivity increased further, and the Rct_avg_ value decreased significantly due to the synergistic effect between MWCNT-COOH, PAA_DES_, and AuNPs (20.30 ohm). Electrochemical characterization results indicated that the electrode with the best electrochemical performance and conductivity was SPCE/MWCNT-COOH/PAA_DES_/AuNP. Therefore, these electrodes were used in the preparation of AFP biosensors.

### 3.5. Electrochemical characterizations of AFP biosensors

**Figure 4:**
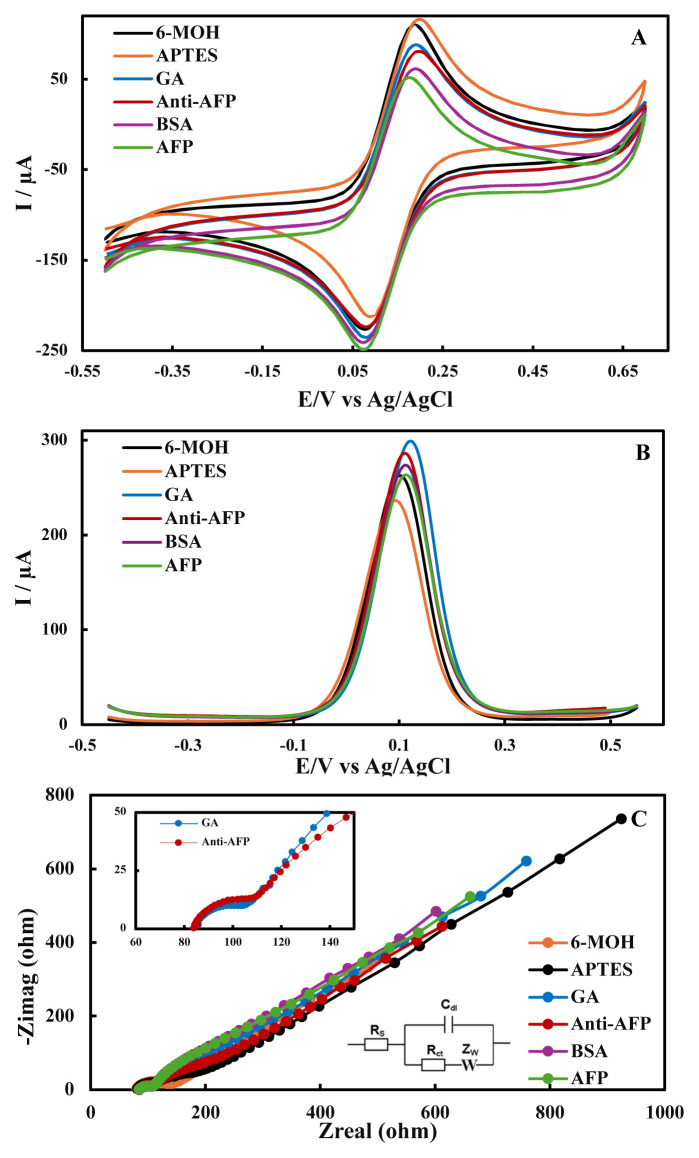
CVs (A), DPVs (B), and EISs (C) of the SPCE/MWCNT-COOH/PAA_DES_/AuNP/Anti-AFP/BSA/AFP immunosensors at each fabrication stage.

For the electrochemical characterization of AFP biosensors prepared using SPCE/MWCNT-COOH/PAA_DES_/AuNPs, measurements were performed using CV, DPV, and EIS methods after each preparation stage (in redox probe). CVs, DPVs, and EISs are presented in Figure 4A-C. Ipa_avg_ and Rct_avg_ are listed in Table S2. After 6-MOH treatment of the AFP biosensors, −OH groups were formed on the surface [72], and the Ipa_avg_ values were 95.0 μA (CV) and 225.1 μA (DPV), while the Rct_avg_ value was 186.3 Ω. In the next step, the −OH groups were treated with APTES, resulting in the formation of siloxane bonds at one end and of amine groups at the other end [73]. As a result, the Rct_avg_ value (107.2 ohms) decreased, while the Ipa_avg_ values increased (CV: 103.3 μA and DPV: 331.9 μA). Then, the amine group of APTES was cross-linked to the aldehyde group of GA, forming a stable, long-chain structure for the immobilization of the antibody on the WE. After immobilization of anti-AFP to the surface, the electrode surface became insulating, and a decrease in Ipa_avg_ and an increment in Rct_avg_ values were observed according to the APTES step. This result demonstrates that Anti-AFP was successfully immobilized on the WE [18,74]. In the BSA and AFP stage, there was a decrease in Ipa_avg_ and an increase in Rct_avg_ as the WE became increasingly insulating. The electrochemical characterization results indicate that AFP biosensors were successfully prepared.

### 3.6. Optimization studies of the AFP biosensors

**Figure 5:**
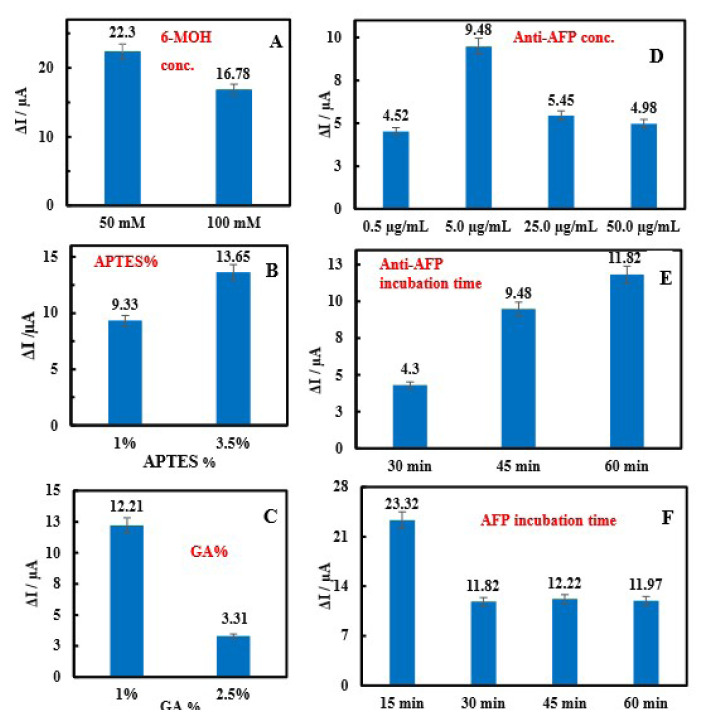
Optimization study of 6-MOH concentration (A), APTES concentration (B), GA concentration (C), anti-AFP concentrations (D), anti-AFP incubation time (E), and AFP incubation time (F) ΔI vs. AFP concentration plots.

Optimization studies during biosensor development are crucial to enhancing biosensor performance. Therefore, the most critical steps in preparing the biosensor were optimized in detail. These studies included the optimization of the amounts of 6-MOH, APTES, GA, and anti-AFP, and of the incubation times for anti-AFP and AFP. The biosensors prepared in the optimization studies were incubated with 1 ng/mL AFP. DPV measurements were performed in the redox probe solution during the BSA and AFP steps. ΔIs (ΔI = I_AFP_- I_BSA_) were calculated using Ipa_avg_. The amounts of 6-MOH, APTES, GA, and anti-AFP as a function of ΔIs, and the incubation graphs of anti-AFP and AFP, are presented in Figure 5A-F. According to Figure 5A, ΔI at 50 mM 6-MOH, whereas ΔI at 100 mM 6-MOH is 16.78 μA. The ΔI value of 50 mM 6-MOH concentration was selected as optimum because it has a higher ΔI value [75]. In the graph in Figure 5B, the ΔI values obtained for 1% and 3.5% APTES are 9.33 μA and 13.65 μA, respectively. The ΔI for 3.5% APTES was determined to be optimal because it was larger [75–76]. In Figure 5C, the ΔI values obtained for 1% and 2.5% GAs percentage during optimization of the GA percentage were 12.21 μA and 3.31 μA, respectively. A GA percentage of 1% was determined to be optimal because it exhibited a higher current response [75–76]. In the anti-AFP concentration optimization study, the ΔI values for anti-AFP concentrations of 0.5 μg mL^−1^, 25.0 μg mL^−1^, and 50.0 μg mL^−1^ were 4.52 μA, 5.45 μA, and 4.98 μA, respectively, and were quite close to each other (Figure 5D). For an anti-AFP concentration of 5.0 μg mL^−1^, the ΔI value was 9.48 μA. The highest current response to the results was obtained with biosensors prepared using an anti-AFP concentration of 5 μg mL^−1^, which was determined to be the optimum. During optimization of the anti-AFP incubation time, ΔI values were determined to be 4.30 μA, 9.48 μA, and 11.82 μA for incubation times of 30, 45, and 60 min, respectively (Figure 5E). The ΔI values indicate that a 30-minute anti-AFP incubation was insufficient for antibody-antigen interaction in the prepared biosensors. The ΔI values of biosensors prepared with 45-minute and 60-minute anti-AFP incubation times were similar. However, because the highest current response was observed in biosensors prepared using a 60-minute anti-AFP incubation, this was determined to be the optimum time. During optimization of the AFP incubation time, the ΔI values at 15, 30, 45, and 60 min were 23.32 μA, 11.82 μA, 12.22 μA, and 11.97 μA, respectively (Figure 5F). The ΔI values of the biosensors prepared with AFP incubation times of 30, 45, and 60 min are very similar and are lower than those at 15 min. The ΔI values of the biosensors prepared with a 15-minute AFP incubation time exhibited the highest current response among all incubation times, and this incubation time was selected as optimal.

### 3.7. Analytical performance of the AFP biosensors

**Figure 6:**
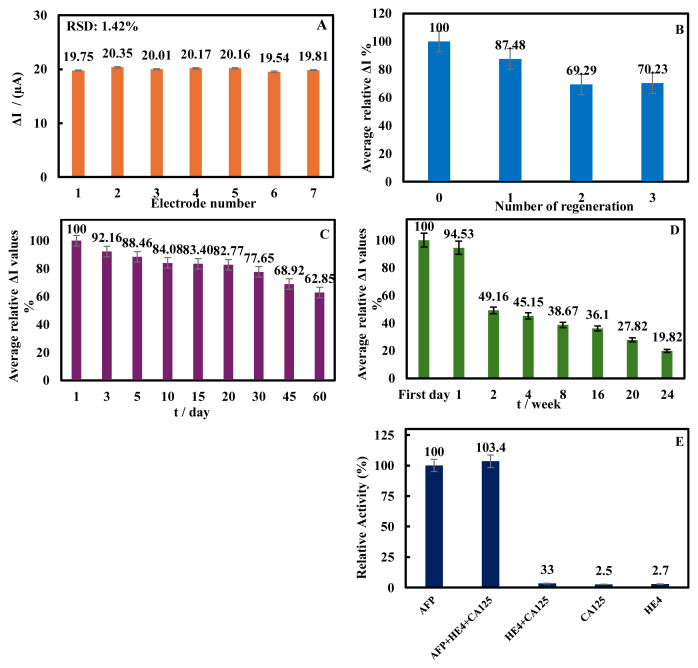
Reproducibility study (A), Reusability study (B), Stability study (C), Shelf life study (D), and Selectivity study (E).

To evaluate the analytical performance of the produced AFP biosensors, AFP analysis was carried out using the DPV method with SPCE/MWCNT-COOH/PAA_DES_/AuNP/Anti-AFP/BSA biosensors in the linear range of 0.5–250 ng mL^−1^ in the redox probe solution. Table shows that ΔI increases with AFP concentration. The sensitivities and detection limits (based on 3 σ/m) of the AFP biosensors were calculated and found to be 0.24 μA mL ng^−1^ and 0.15 ng mL^−1^, respectively [18]. The repeatability of the AFP biosensors was tested using an AFP solution (25 ng mL^−1^) within the detection range, and consistent results (% RSD: 4.80%) were obtained across five tests. Seven biosensors were fabricated and used for AFP measurement (25 ng mL^−1^) on different days for reproducibility (Figure 6A). The RSD% of response changes among different biosensors was 1.42%, indicating that the biosensors produced consistent results. Evaluating the biosensor regeneration is crucial for assessing surface stability and rebinding ability after dissociation of the antibody-antigen immune complex [76]. AFP biosensors were treated with a 10 mM HCl solution for 2 min and then assessed for reusability. They were then incubated once more with AFP (25 ng mL^−1^), followed by DPV analysis. AFP biosensors showed signal decreases of 12.54% and 30.71% after the first and second regenerations, respectively (Figure 6B). During the third regeneration, the biosensors’ response increased by 29.77%, so the regeneration study was terminated because the expected decrease did not occur. These findings demonstrate that the disposable biosensors can be reused after regeneration.

To test stability, AFP biosensors were prepared, and AFP (25 ng mL^−1^) assays were performed for 60 days. Mean relative ΔI% values were calculated relative to the baseline value and plotted against days (Figure 6C). The response of the AFP biosensors decreased relative to baseline by 7.84% after 3 days, 11.54% after 5 days, 17.23% after 20 days, and 22.35% after 30 days compared to the baseline value. Although the signal of the AFP biosensors decreased by 37.15% at the end of day 60, it continued to indicate the presence of the AFP antigen. To examine the shelf life of AFP biosensors, eight biosensors were produced through the BSA stage and stored at +4 °C until week 32. AFP (25 ng mL^−1^) analyses were performed using AFP biosensors at weeks 1, 2, 4, 8, 16, 20, and 24. Using the analysis results, mean relative ΔI% values were calculated relative to the initial value and plotted against the number of weeks (Figure 6D). The response of AFP biosensors decreased by 47%, 50.84%, 61.33%, and 72.18% after 1, 2, 8, and 20 weeks of storage, respectively, compared to the initial value. AFP analysis was not performed for the 32-week storage period because an 80.18% signal decrease was observed after 24 weeks. One of the most important factors affecting the stability and shelf life of biosensors is the material used to modify the electrode surface. The MWCNT-COOH used in this study is a graphene-based material whose structure is resistant to degradation. PAAs and AuNPs are known for their biocompatibility. In this study, the combined use of MWCNT-COOH, PAA_DES_, and AuNPs is thought to increase the stability and shelf life of the AFP biosensors. The responsiveness of AFP biosensors to other cancer biomarkers, CA125 and human epididymis protein 4 (HE4), was investigated. For this, CA125 (50 pg mL^−1^), HE4 (50 pg mL^−1^), and AFP solutions (25 ng mL^−1^) were prepared separately, along with CA125-HE4 solution (50 pg mL^−1^ each) and CA125-HE4-AFP solution (HE and CA125: 50 pg and AFP: 25 ng mL^−1^). AFP biosensors were incubated with these antigen solutions, and DPV measurements were recorded in the redox probe solution. This test was conducted to determine whether these antigens caused interference. The response of AFP biosensors to CA125, HE4, a mixture of CA125 and HE4, and a mixture of CA125, AFP, and HE4 was 3.5% (Figure 6E). These results demonstrated that AFP biosensors had good selectivity for AFP in the presence of other cancer markers.

The analytical performance of various label-free electrochemical AFP biosensors is presented in Table S3. The sensitivity values of AFP immunosensors reported in the literature generally range from 0.183 to 0.247 μA ng^−1^ mL. The sensitivity of the sensor we developed is 0.2372 μA ng^−1^ mL, which demonstrates a high and competitive analytical performance compared to existing studies. Furthermore, considering that serum AFP levels are 25 ng mL^−1^, while some sensors in the literature have linear ranges at very low limits (e.g., 0.05–100 pg mL^−1^ or 0.001–10 ng mL^−1^), some studies have high upper limits but low detection limits that are not clinically adequate. The linear range (0.5–250 ng mL^−1^) obtained in our study encompasses clinical serum AFP concentrations and offers a practical advantage. The AFP biosensors used in this study accurately determined AFP levels in human serum, yielding high recoveries. The developed AFP biosensors are suitable for POCT, cost-effective, and capable of providing practical measurements of AFP levels without requiring expert assistance. The AFP biosensors we developed are considered promising for the early diagnosis of various cancers.

### 3.8. AFP analysis in the human serum

AFP biosensors were prepared under optimal operating conditions for the analysis of real samples and were utilized to detect AFP in commercial human serum. For this purpose, human serum samples were prepared by adding a known amount (25 ng mL^−1^) of AFP to commercial human serum samples diluted at a 1:100 ratio [18,21,44]. Serum samples containing AFP antigen were incubated with the developed immunosensors, and AFP analyses were performed. Using Ipa_avg_ values, %recovery and %error values were calculated at 97.80% and 2.20%, respectively. Analysis of AFP in commercial human serum was completed in two minutes using AFP biosensors, without requiring lengthy procedures. These results suggest that more sensitive and selective biosensors can be developed in the future for the analysis of other targeted cancer biomarkers. Disposable, label-free AFP biosensors are candidates for use in POCT.

## 4. Conclusion

Multiple studies on AFP biosensors have been conducted in recent years. Some of these studies use labeled biosensors that employ a second antibody and signal enhancers. This approach presents cost and time disadvantages. Most label-free electrochemical AFP biosensors reported in the literature use three separate electrode systems and do not allow on-site analysis. The SPCEs we used in our study are disposable, rapid, practical, and suitable for point-of-care testing. SPCEs can be carried anywhere, making them suitable for portable analysis.

In this study, label-free, practical, POCT-candidate electrochemical AFP biosensors were developed for the determination of AFP, an FDA-approved cancer biomarker. The surfaces of SPCEs were first modified with MWCNT-COOH to increase electrode conductivity and electrochemical activity. PAA, a conductive redox polymer, was prepared in DES for the first time in our study and deposited onto MWCNT-COOH-modified SPCEs. π-π electronic and hydrophobic interactions between PAA_DES_ and MWCNT-COOH further increased the electronic conductivity, demonstrating a synergistic effect. The formation of AuNPs on SPCEs modified with MWCNT-COOH/PAADES further increased electronic conductivity. The combined use of these three materials exhibited a synergistic effect, thereby increasing the sensitivity, stability, and shelf life of the AFP biosensors. Positive findings were also obtained in the regeneration study. Although disposable, the AFP biosensors exhibite high repeatability and reproducibility. In the presence of other cancer markers (CA125 and HE4), the AFP biosensors demonstrated high selectivity for AFP over these markers. It was used for AFP analysis in human blood serum samples, and a recovery exceeding 98% was achieved. The direct detection of the AFP antigen, characterized by a wide linear range and a low detection limit, suggests that more sensitive and selective biosensors can be developed to detect other cancer biomarkers.

## Supplementary material

### 1. Experimental

#### 1.1. Reagents

Azur A (AA), chloroauric acid (HAuCl_4_), bovine serum albumin (BSA), potassium hexacyano ferrate (K_3_Fe(CN)_6_), potassium hexacyano ferrite (K_4_Fe(CN)_6_, glutaraldehyde (GA), human serum (from male AB coagulated whole blood, H6914), 6-mercapto hexanol (6-MOH), choline chloride ([(CH_3_)_3_NCH_2_CH_2_OH]Cl) were obtained from Sigma-Aldrich (USA). Anti-AFP and AFP were purchased from Novus Biologicals USA. 3-aminopropyltriethoxysilane (APTES), ethylene glycol (C_2_H_6_O_2_), dimethyl formamide (DMF), carboxyl-functionalized multi-walled carbon nanotube (MWCNT-COOH), potassium chloride (KCl), potassium dihydrogen phosphate (KH_2_PO_4_), potassium hydrogen phosphate (K_2_HPO_4_) and hydrochloric acid (HCl) It was supplied by Merck. K_2_HPO_4_, KH_2_PO_4_, and KCl were used to prepare phosphate buffer solution (PBS). All solutions were prepared with ultrapure water (Millipore, 18MΩ cm).

#### 1.2. Instruments

Voltammetric analysis was performed using a Bipotentiostat/Galvanostat μStat 400 from Metrohm DropSens (Oviedo, Spain), using DropView 800 software on a computer. Electrochemical impedance spectroscopy (EIS) measurements were performed on a Gamry Potentiostat/Galvanostat, Reference 1010E (Gamry Instruments, Warminster, USA), running EChem Analyst. Disposable screen-printed carbon electrodes (SPCEs) were obtained from Metrohm DropSens (Oviedo, Spain; reference: DRPX1110) and have an integrated three-electrode system structure consisting of working (WE) and counter electrodes made of carbon and a reference electrode made of silver. The chemical characterization of the modified SPCEs was carried out using Perkin Elmer Fourier transform infrared (FT-IR) and Panalytical Empyrean X-ray Diffraction (XRD) and morphological characterization was carried out using ZEISS Gemini 1 Field Emission Scanning Electron Microscope (FE-SEM).

#### 1.3. Electrochemical measurements

Electrochemical characterizations and AFP analysis were performed in redox probe solution (FF: 5 mM K_3_Fe(CN)_6_/K_4_Fe(CN)_6_ in 1 M KCl). EIS measurements were performed in the frequency range of 50.000 Hz - 0.05 Hz. DPV measurements were performed with the following parameters: Epulse: 70 mV, tpulse: 0.1 s, Estep: 5 mV, scan rate: 5 mV s^−1^. The following parameters were used for CV measurements: potential ranges between −0.5 V and +0.8 V at 50 mV s^−1^.

#### 1.4. Preparation of MWCNT-COOH and AADES

1 mg of MWCNT-COOH and DMF:H_2_O (1:1 ratio) were stirred in a sonicator for 3 hours, resulting in MWCNT-COOH suspension [1]. To produce the DES solution, ethylene glycol and choline chloride (in a 1:2 molar ratio) were heated in a sealed glass flask at 80 °C with stirring until the mixture became transparent. The AA solution (1 mM) was produced in a mix of 90% DES and 10% PBS (50 mM, pH 8.0, 0.1 M KCl/KNO_3_) [2,3].

#### 1.5. Preparation of the modified electrode

MWCNT-COOH suspension (3 μL) was deposited onto the working electrode (WE) surfaces of SPCEs by the layer-by-layer (LBL) method and left to dry (SPCE/MWCNT-COOH). Then, the electropolymerization of AA_DES_ monomer on SPCE/MWCNT-COOHs (−0.8 V to +1.0 V, 100 mV s^−1^) was carried out by applying 20 cycles of CV [4,5]. The AuNP modification was applied to the modified electrode (SPCE/MWCNT-COOH/PAA_DES_), as in our previous studies [4,6–7]. For this, 10 cycles of CV were conducted within the potential range of −1.3 to −0.2 V utilizing a 4 mM HAuCl_4_ solution.

#### 1.6. Fabrication of the AFP biosensors

The first and most crucial step in preparing biosensors is the immobilization of antibodies. In this study, SAM layers were first created to immobilize anti-AFP. To create SAM layers, 3 μL of 50 mM 6-MOH was placed on the surface of SPCE/MWCNT-COOH/PAA_DES_/AuNPs and left to sit overnight [8]. Then, to treat with the silane agent, 3 μL of 3.5% APTES solution was applied to the WE and left overnight [9]. Thus, SAM layers were formed on the modified electrode surface. 3 μL of 1% GA solution was applied onto the APTES-modified electrodes and left for 15 min. 3 μL of antibody solution (50 μg mL^−1^ Anti-AFP) was dropped onto the surfaces of GA-activated electrodes and left for 30 minutes [10]. Then, 3 μL of 1% BSA solution was dropped and left for 30 minutes. Finally, 3 μL of AFP solution was treated with the WE and left for 15 min. Thus, the preparation process of AFP biosensors has been completed. The preparation scheme is given in Figure 1A-C.

Figure S1 CVs of the different SPCE/MWCNT-COOH layers (0 LBL, 1 LBL, 2 LBL, 3 LBL and 4 LBL) SPCE/MXene-COOH in 5 mM redox probe solution at different scan rates (10-25-50-75-100-125 mV s^−1^) (A, B, C, D, E), Ipa-*v*^1/2^ graphs (F, G, H, I, J).

Figure S2 CVs of 20 cycles (A), 25 cycles (B), and 30 cycles (C) of electropolymerization of AA_DES_ on SPCE/MWCNT-COOH

Figure S3 CVs of the different cycle number PAA_DES_ modified SPCE/MWCNT-COOH in redox probe solution at different scan rates (10-25-50-75-100-125 mV s^−1^) (A, B, C), Ipa-*v*^1/2^ graphs (D, E, F).

**Table S1:** Ipa_avg_ and Rct_avg_ values obtained from CVs, DPVs, and EIS of SPCE, SPCE/PAA_DES_, SPCE/AuNP, SPCE/MWCNT-COOH, SPCE/MWCNT-COOH/PAA_DES_, SPCE/MWCNT-COOH/PAA_DES_/AuNP

Formulation	CV Ipa _avg_ (μA)	DPV Ipa _avg_ (μA)	EIS Rct _avg_ (ohm)
**SPCE**	128.62	147.89	109.00
**SPCE/PAA** **_DES_**	154.21	173.65	76.70
**SPCE/AuNP**	157.05	174.74	61.73
**SPCE/MWCNT-COOH**	159.79	172.65	229.20
**SPCE/MWCNT-COOH/PAA** **_DES_**	162.59	177.00	213.90
**SPCE/MWCNTCOOH/PAA** **_DES_** **/AuNP**	164.66	187.17	20.30

**Table S2:** Ipa_avg_ and Rct_avg_ values obtained from CVs, DPVs, and EIS of AFP immunosensors fabricated with SPCE/MWCNT-COOH/PAA_DES_/AuNP

Formulation	CV Ipa _avg_ (μA)	DPV Ipa _avg_ (μA)	EIS Rct _avg_ (ohm)
**6-MOH**	95.0	225.1	186.3
**APTES**	103.3	331.9	107.2
**GA**	77.6	243.6	26.1
**Anti-AFP**	73.4	256.1	34.9
**BSA**	64.9	233.6	60.6
**AFP**	56.0	221.8	68.3

**Table S3:** Analytical performances of different label-free electrochemical AFP immunosensors

Formulation	LOD	Detection range	Sensitivity	Method	Ref.
GCE/Thi/Chit/HRP/AuNP/AntiAFP/BSA/AFP	0.03 pg mL ^−1^	0.10 -10000 pg mL ^−1^	5.82 pg mL ^−1^	CV	[ 11]
GCE/MCHS@MnO _2_/AntiAFP/BSA/AFP	0.03 ng mL ^−1^	0.10–420 ng mL ^−1^	0.21 ng mL ^−1^	SWV	[ 12]
GCE/isoorientin/AntiAFP/BSA/AFP	0.0002 ng mL ^−1^	0.001–10 ng mL ^−1^	0.2 pg mL ^−1^	DPV	[ 13]
SPCE/RGO/PNR/AuNP/AntiAFP/BSA/AFP	0.79 ng mL ^−1^ 0.86 ng mL ^−1^	1–500 ng mL ^−1^	0.247 μA ng ^−1^ mL 0.183 μA ng ^−1^ mL	DPV SWV	[ 14]
GCE/3D-CuFC-C/AntiAFP/BSA/AFP	1.40x10 ^−8^ ng mL ^−1^	2.25x10 ^−8^–2.25x10 ^2^ ng mL ^−1^	2.10 μA ng ^−1^ mL	CV	[ 15]
GCE/CBNP@ PdNP/AntiAFP/BSA/AFP	0.0039 ng mL ^−1^ 0.0131 ng mL ^−1^	0.005–1000 ng mL ^−1^	39.03 Rct ng ^−1^ mL	SWV EIS	[ 16]
GCE/RGO@PtNPs/MoS _2_/AntiAFP/BSA/AFP	0.12 pg mL ^−1^	1–10 ^5^ pg mL ^−1^	35.075 pg mL ^−1^	DPV	[ 17]
ITO/3-TMSPM/AntiAFP/BSA/AFP	0.01 pg mL ^−1^	0.05–100 pg mL ^−1^	179.09 pg mL ^−1^	EIS	[ 18]
GCE/NS-CDs/Fe _2_O _3_@NS-CDs/AntiAFP/BSA/AFP	16.8 fg mL ^−1^	5–1.0x10 ^4^ pg mL ^−1^	4.33 pg mL ^−1^	DPV	[ 19]
**SPCE/MWCNT-COOH/PAA** **_DES_** **/AuNP/AntiAFP/BSA/AFP**	**0.15 ng mL** **^−1^**	**0.5–250 ng mL** **^−1^**	0.2372 μA ng ^−1^ mL	**DPV**	**This work**

3 D-CuFC-C: Cu_2_[Fe(CN)_6_]-C nanocrystals
3-TMSPM:3-(Trimethoxysilyl)propyl methacrylate
AuNP: Gold nanoparticles
BSA: Bovin serum albumin
CBNP: Carbon black nanoparticles
Chit: Chitosan
CV: Cyclic voltammetry
DPV: Differantiel puls voltammetry
EIS: Electrochemical empedans spectroscopy
Fe_2_O_3_@NS-CDs: Fe_2_O_3_ doped carbon dots
GCE: Galssy carbon electrode
HRP: Horseradish peroxidase
ITO: Indium tin oxide
MCHS: Mesoporous carbon hollow sphere
MnO_2_: Manganese dioxide
MWCNT-COOH: Carboxyl-functionalized multi-walled carbon nanotube
NS-CDs: Nitrogen-Sulfur-doped carbon dots
PAA_DES_: PolyAzur A (deep eutectic solvent)
PdNP: Palladium nanoparticles
PNR: Poly-neutral red
RGO: Reduce graphene oxide
SWV: Square Wave voltammetry
Thi: Thionine

References1

ZhangZ
XuS
WuY
ShiS
XiaoG

Recent advances of pervaporation separation in dmf/h2o solutions: A review
Membranes
2021
11
6
455
10.3390/membranes11060455
34203059
PMC82345232

YılmazM
BilgiM

A disposable impedimetric immunosensor for the analysis of CA125 in human serum samples
Biomed Microdevices
2024
26
1
8
10.1007/s10544-023-00691-x
38180587
3

AticiT
Bilgi KamaçM
YilmazM
Yılmaz KabacaA

Zinc oxide nanorod/polymethylene blue (deep eutectic solvent)/gold nanoparticles modified electrode for electrochemical determination of serotonin (5-HT)
Electrochim Acta
2023
458
142484
10.1016/j.electacta.2023.142484
4

Jiménez-FiérrezF
González-SánchezM
Jiménez-PérezR
IniestaJ
ValeroE

Glucose biosensor based on disposable activated carbon electrodes modified with platinum nanoparticles electrodeposited on poly(Azure A)
Sensors
2020
20
16
4489
10.3390/s20164489
32796638
PMC74721695

DongS
ChuQ

Study of the electrode process of hemoglobin at a polymerized azure A film electrode
Electroanalysis
1993
5
2
135
140
10.1002/elan.1140050207
6

BilgiM
AyranciE

Biosensor application of screen-printed carbon electrodes modified with nanomaterials and a conducting polymer: ethanol biosensors based on alcohol dehydrogenase
Sensors and Actuators B: Chemical
2016
237
849
855
10.1016/j.snb.2016.06.164
7

Bilgi KamaçM
AltunM
YılmazM
Yılmaz AktanA
AktanS


Point-of-care testing: a disposable label-free electrochemical CA125 and HE4 immunosensors for early detection of ovarian cancer
Biomedical Microdevices
2023
25
2
18
10.1007/s10544-023-00659-x
37140852
8

VuralB
ÇalışkanM
Bilgi KamaçM
SezgintürkMK

A disposable and ultrasensitive immunosensor for HE4 detection
Chemical Papers
2024
1
12
10.1007/s11696-024-03359-9
9

YaşarZG
YaşarÜ
KahramanO
UlusalF
Gönülİ


An APTES-Coated magnetic nanoparticle–based immunosensor for prostate cancer diagnosis
Journal of Molecular Structure
2026
146192
10.1016/j.molstruc.2026.146192
10

ŞimşekÇ
Sonuç KaraboğaMN
SezgintürkMK

A new immobilization procedure for electrochemical immunosensor development
Talanta
2015
144
210
218
10.1016/j.talanta.2015.06.010
26452812
11

LuD
XuQ
PangG
LuF

A novel electrochemical immunosensor for ultrasensitive α-fetoprotein detection
Biomedical Microdevices
2018
20
1
11
10.1007/s10544-018-0291-7
2986900112

ZhuX
DaiY
SunY
LiuH
SunW


Rapid fabrication of electrode for the detection of alpha fetoprotein based on MnO2 functionalized mesoporous carbon hollow sphere
Materials Science and Engineering C
2020
107
110206
10.1016/j.msec.2019.110206
31761168
13

ShiP
XieR
WangP
LeiY
ChenB


Non-covalent modification of glassy carbon electrode and application to alpha-fetoprotein immunosensor
Sensors and Actuators B: Chemical
2020
305
127494
10.1016/j.snb.2019.127494
14

ErdemK
Bilgi KamaçM

Kanser biyobelirteci alfa-fetoproteinin elektrokimyasal tayini için tek kullanımlık etiketsiz yeni AFP immünosensörünün geliştirilmesi
Journal of the Institute of Science and Technology
2021
11
2
1279
1292
(in Turkish)
15

GuoJ
WangJ
WangZ
LiS
WangJ

High sensitivity alpha-fetoprotein immune-electrochemical detection using CuFC-C nanocrystals
Biosensors and Bioelectronics
2022
218
114766
10.1016/j.bios.2022.114766
36206667
16

OlorundareF
SipukaD
SebokolodiT
KodamaT
ArotibaO


An electrochemical immunosensor for an alpha-fetoprotein cancer biomarker
Analytical Methods
2023
15
29
3577
3585
10.1039/d3ay00552a
37458385
17

ZhangS
ChenX
HuS
CaiK
PengC


Electrochemical immunosensor based on PtNPs/MoS2@rGO composite for alpha-fetoprotein detection
Microchimica Acta
2024
191
11
662
39387898
10.1007/s00604-024-06712-718

ÖzcanB
ÖzayH
ÖzayÖ
SezgintürkMK

Early detection of ovarian cancer with a disposable immunosensor platform
Microchemical Journal
2024
205
111340
10.1016/j.microc.2023.111340
19

TuoY
XuR
GuanY
LiS
YangH


Construction of electrochemical immunosensor integrating doped carbon dots
Talanta
2025
291
127887
10.1016/j.talanta.2025.127887
40054220


## Figures and Tables

**Table:** Calibration parameters and analytical figures of merit of the developed electrochemical sensor

Linear range (ng mL^−1^)	Calibration equation	m (μA ng^−^^1^ mL)	n (*μA*)	r	LOD (ng mL^−1^)	LOQ (ng mL^−1^)
0.5–250	y = 0.2372x + 5.5053	0.2372	5.5053	0.9987	0.15	0.50
