# Supplementary figures and images for: Electrochemical biosensing of alpha-fetoprotein based on carboxylated multi-walled nanotube-polyAzur A (in DES)-gold nanoparticles
Source: Turk J Chem. 2026 Apr 10;50(3):271–84. doi: 10.55730/1300-0527.3797 (PMC13384571; doi:10.55730/1300-0527.3797)

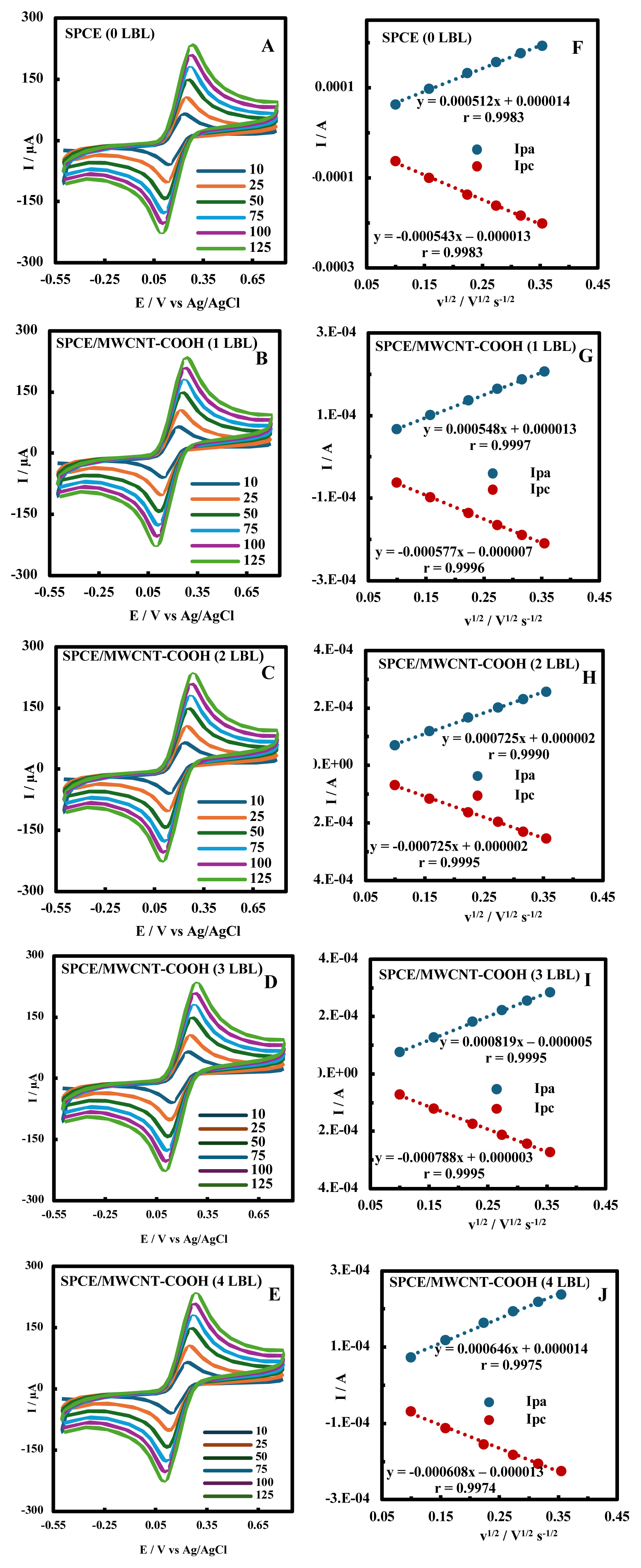

Supplement: Figure S1 — CVs of the different SPCE/MWCNT-COOH layers (0 LBL, 1 LBL, 2 LBL, 3 LBL and 4 LBL) SPCE/MXene-COOH in 5 mM redox probe solution at different scan rates (10-25-50-75-100-125 mV s−1) (A, B, C, D, E), Ipa-v1/2 graphs (F, G, H, I, J). [file tjc-50-03-271s1.tif]

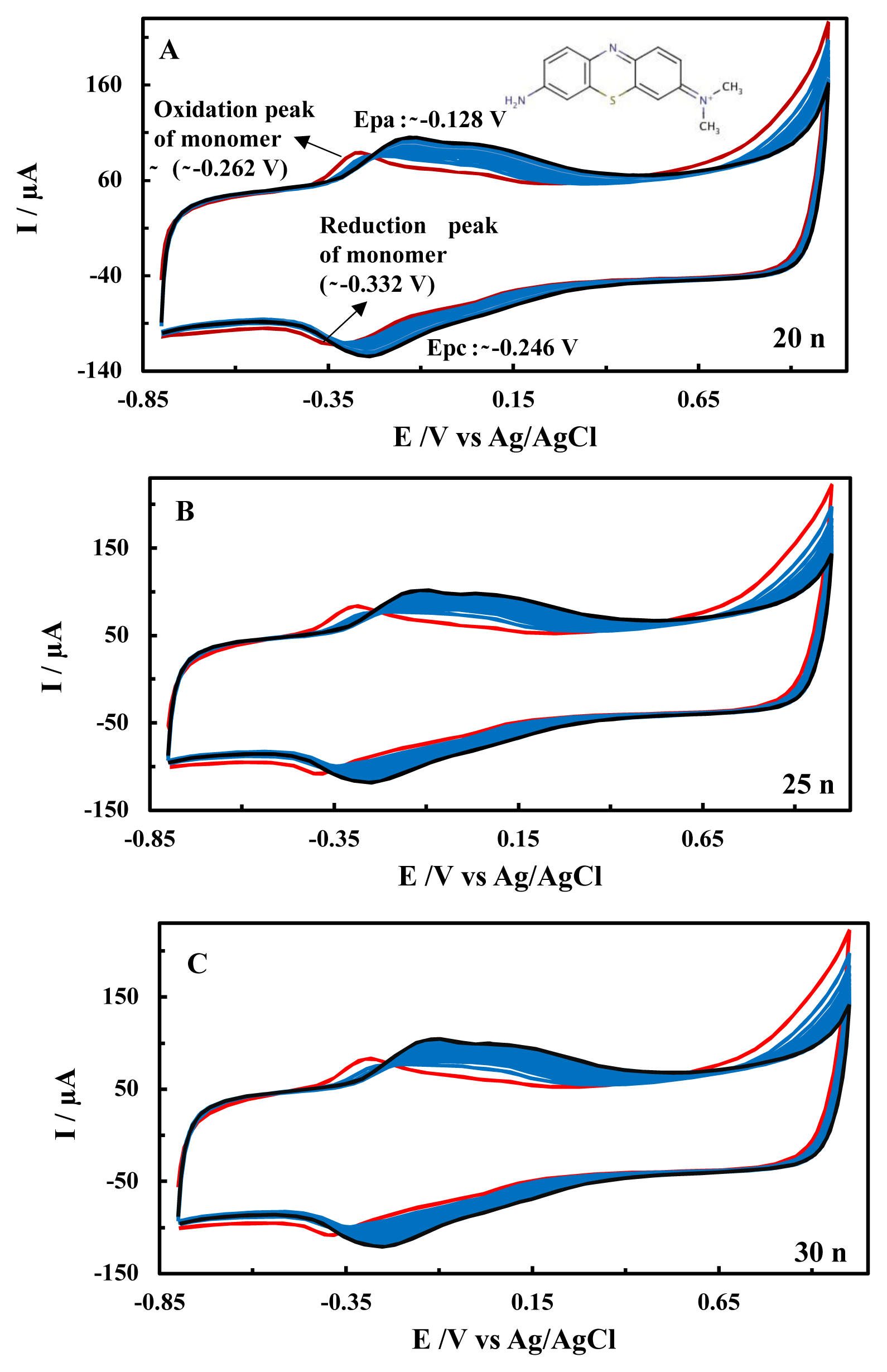

Supplement: Figure S2 — CVs of 20 cycles (A), 25 cycles (B), and 30 cycles (C) of electropolymerization of AADES on SPCE/MWCNT-COOH [file tjc-50-03-271s2.tif]

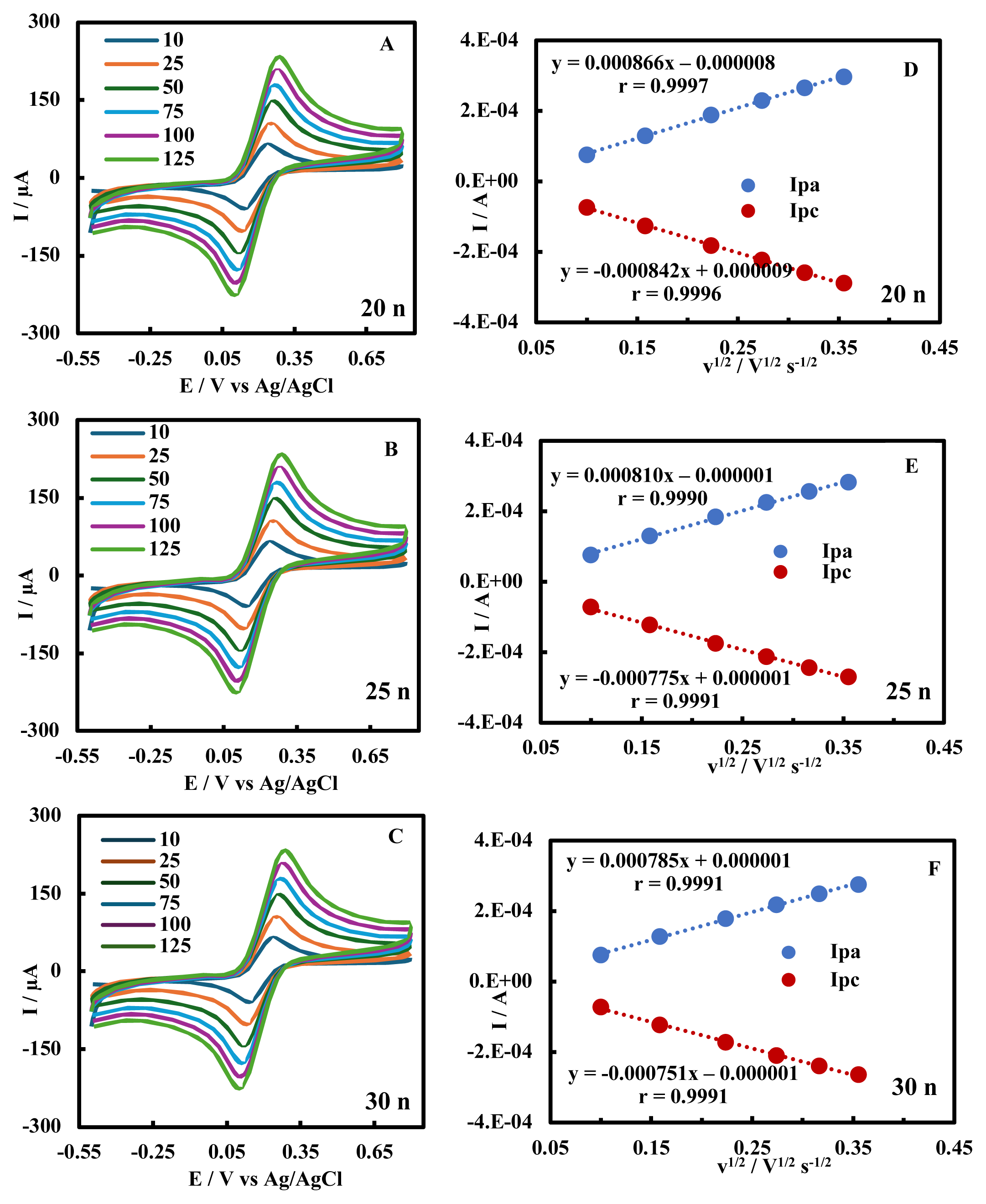

Supplement: Figure S3 — CVs of the different cycle number PAADES modified SPCE/MWCNT-COOH in redox probe solution at different scan rates (10-25-50-75-100-125 mV s−1) (A, B, C), Ipa-v1/2 graphs (D, E, F). [file tjc-50-03-271s3.tif]
